# Acarbose suppresses symptoms of mitochondrial disease in a mouse model of Leigh Syndrome

**DOI:** 10.1093/geroni/igaa057.3271

**Published:** 2020-12-16

**Authors:** Alessandro Bitto, Anthony Grillo, Matt Kaeberlein

**Affiliations:** University of Washington, Seattle, Washington, United States

## Abstract

Mitochondrial diseases are pathologies characterized by impairment in mitochondrial function. Mitochondrial dysfunction is also a hallmark of the aging process. Rapamycin, a drug that increases lifespan and reduces the incidence of age-related pathologies in multiple models, increases survival and reduces the impact of neurological symptoms in a mouse model lacking the complex I subunit Ndufs4. Here we show that acarbose, another drug that extends lifespan in mice, suppresses symptoms of disease and improves survival of Ndufs4-/- mice. Unlike rapamycin, acarbose rescues disease phenotypes independently of mTOR inhibition. Furthermore, rapamycin and acarbose have additive effects on clasping and maximum lifespan in Ndufs4-/- mice. Acarbose rescues mitochondrial disease independently of glycolytic flux and Sirt3 activity by potentially remodeling the microbiome. This study provides the first evidence that the microbiome may rescue severe mitochondrial disease and proof of principle that biological aging and mitochondrial disorders are driven by common mechanisms.

